# Anatomical Variation of Selected Cranial Emissary Foramina in Adult Greek Skulls: A Mixed Anatomical and Computed Tomography Study

**DOI:** 10.7759/cureus.110212

**Published:** 2026-06-03

**Authors:** Michael Kostares, Evangelos Kostares, George Triantafyllou, George Tsakotos, Nikos Pantazis, Dimitrios Filippou, Theano Demesticha, Panagiotis Papanagiotou, Theodore Troupis, Maria Piagkou

**Affiliations:** 1 Anatomy, School of Medicine, Faculty of Health Sciences, National and Kapodistrian University of Athens, Athens, GRC; 2 Oral and Maxillofacial Surgery, “Evangelismos” General Hospital, National and Kapodistrian University of Athens, Athens, GRC; 3 Hygiene, Epidemiology and Medical Statistics, School of Medicine, Faculty of Health Sciences, National and Kapodistrian University of Athens, Athens, GRC; 4 Radiology, Aretaieion University Hospital, National and Kapodistrian University of Athens, Athens, GRC

**Keywords:** anatomical variation, cranial emissary foramina, morphology, morphometry, skull base anatomy

## Abstract

Introduction

Cranial emissary foramina (CEF) are variable osseous channels that may transmit emissary veins (EVs) between extracranial venous networks and intracranial dural venous sinuses. Their anatomical relevance depends not only on their presence but also on laterality, size, morphology, and relationships with adjacent cranial landmarks. This study aimed to evaluate the prevalence, laterality, morphology, morphometry, and topographic anatomy of selected CEF in an adult Greek sample using a mixed anatomical and computed tomography (CT)-based design.

Methods

This retrospective observational study included 200 adult specimens, comprising 50 dry skulls (25.0%) and 150 CT examinations (75.0%). The parietal emissary foramina (PEF), sphenoidal emissary foramina (SEF), mastoid emissary foramina (MEF), and posterior condylar canal (PCC) were assessed for presence, laterality, morphology, morphometry, and topographic relationships. Side-to-side variation, co-occurrence patterns, and exploratory associations with selected constant cranial foramina, craniometric parameters, sex, and specimen type were also examined. Interobserver reliability was assessed for continuous measurements.

Results

The PCC was the most frequent structure, identified in 162 specimens (81.0%), followed by the MEF in 157 (78.5%), the PEF in 137 (68.5%), and the SEF in 49 (24.5%). Among specimens in which the corresponding foramen was present, bilateral expression was observed in 64 PEF cases (46.7%), 12 SEF cases (24.5%), 74 MEF cases (47.1%), and 73 PCC cases (45.1%). The SEF showed the highest proportion of unilateral cases, with unilateral expression in 37 cases (75.5%). Morphologic classification showed a predominance of oval forms across the examined foramina. Morphometric profiles differed by anatomical region, with the SEF exhibiting the smallest dimensions and the PCC the largest. Topographic measurements demonstrated distinct regional relationships for each foramen. Co-occurrence analysis identified a selective association between the presence of SEF and PCC. Exploratory regression analyses demonstrated selective associations with skull base length, adjacent foraminal dimensions, cranial breadth, and sex. Interobserver reliability was high to excellent.

Conclusions

CEF were frequent but anatomically heterogeneous structures in this adult Greek sample. Their assessment should be region-specific and should integrate prevalence, laterality, morphometry, morphology, and topographic anatomy. The observed exploratory associations require confirmation in independent anatomical and imaging studies incorporating vascular imaging techniques to distinguish osseous foramina from functionally relevant EV pathways.

## Introduction

Cranial emissary foramina (CEF) are variable osseous openings of the cranial vault and skull base that transmit emissary veins (EVs) between the intracranial dural venous sinuses and extracranial venous networks [1]. Their anatomical relevance extends not only to their presence but also to their size, laterality, morphology, and relationships with adjacent cranial landmarks. In radiological and surgical practice, these structures may be relevant when they are located near the middle cranial fossa, mastoid region, or posterior cranial fossa, particularly because emissary venous channels may be encountered along clinically important skull-base pathways [2].

Various studies have provided important data on the prevalence and morphometry of CEF, but the reported findings vary widely [3-5]. This variability is likely related to both biological and methodological factors, including population differences, study material, anatomical definitions, and measurement protocols. Dry-skull studies, computed tomography (CT), magnetic resonance imaging (MRI), and vascular imaging may emphasize different aspects of the same anatomical region [6]. Therefore, comparisons across studies should take these factors into account.

Although the parietal emissary foramen (PEF), sphenoidal emissary foramen (SEF), mastoid emissary foramen (MEF), and posterior condylar canal (PCC) have each been described in anatomical or imaging studies, they are usually discussed in relation to their specific cranial region. The PEF is mainly related to the posterior cranial vault, whereas the SEF is associated with the greater wing of the sphenoid and the foramina of the middle cranial fossa [7]. The MEF is related to the mastoid and sigmoid sinus region, while the PCC is associated with the occipital condylar region and posterior condylar emissary vein [8]. Comparative assessment of these foramina within the same sample and under a shared morphometric and topographic protocol has been investigated less extensively.

The present study aimed to investigate the prevalence, laterality, morphology, morphometry, and topographic anatomy of four CEF, namely the PEF, SEF, MEF, and PCC, in an adult Greek sample using a mixed anatomical-radiological design. The study also explored side-to-side variation, co-occurrence patterns, and associations with selected constant cranial foramina, craniometric parameters, sex, and specimen type. By combining dry skull material with CT examinations under a common measurement framework, the study sought to determine whether these foramina demonstrate a shared pattern of expression or, instead, region-dependent anatomical variability.

## Materials and methods

Study design and sample

This retrospective observational study used a mixed anatomical and CT-based design to evaluate selected cranial emissary foramina (CEF) in an adult Greek sample. Dry skulls derived from documented osteological collections originating from cemeterial sources in Attica and the Peloponnese, Greece, were assessed between February and November 2020. Dry skull specimens were eligible for inclusion when sex had been documented and no gross deformity, fracture, destructive lesion, or postmortem alteration compromised identification or measurement of the examined structures. CT examinations were retrieved retrospectively from an institutional archive between March and June 2021. CT examinations were eligible when sex was recorded in the institutional archive, image quality permitted assessment of the anatomical regions of interest, and slice thickness was 1 mm or less. Cases with missing required study data or inadequate visualization of the relevant cranial regions were excluded.

Dry skull specimens were assigned non-identifying study codes at collection. CT examinations were fully de-identified before analysis and assigned study-specific codes. The study was approved by the Ethics Committee of the National and Kapodistrian University of Athens, School of Medicine, Athens, Greece (protocol no. 1153/26.01.2026). Acquisition of the osteological material was based on written consent from the collaborating centers, and patient consent for CT examinations was waived because of the retrospective design and the use of fully de-identified archived imaging data.

Study variables and measurement protocol

**Figure 1:**
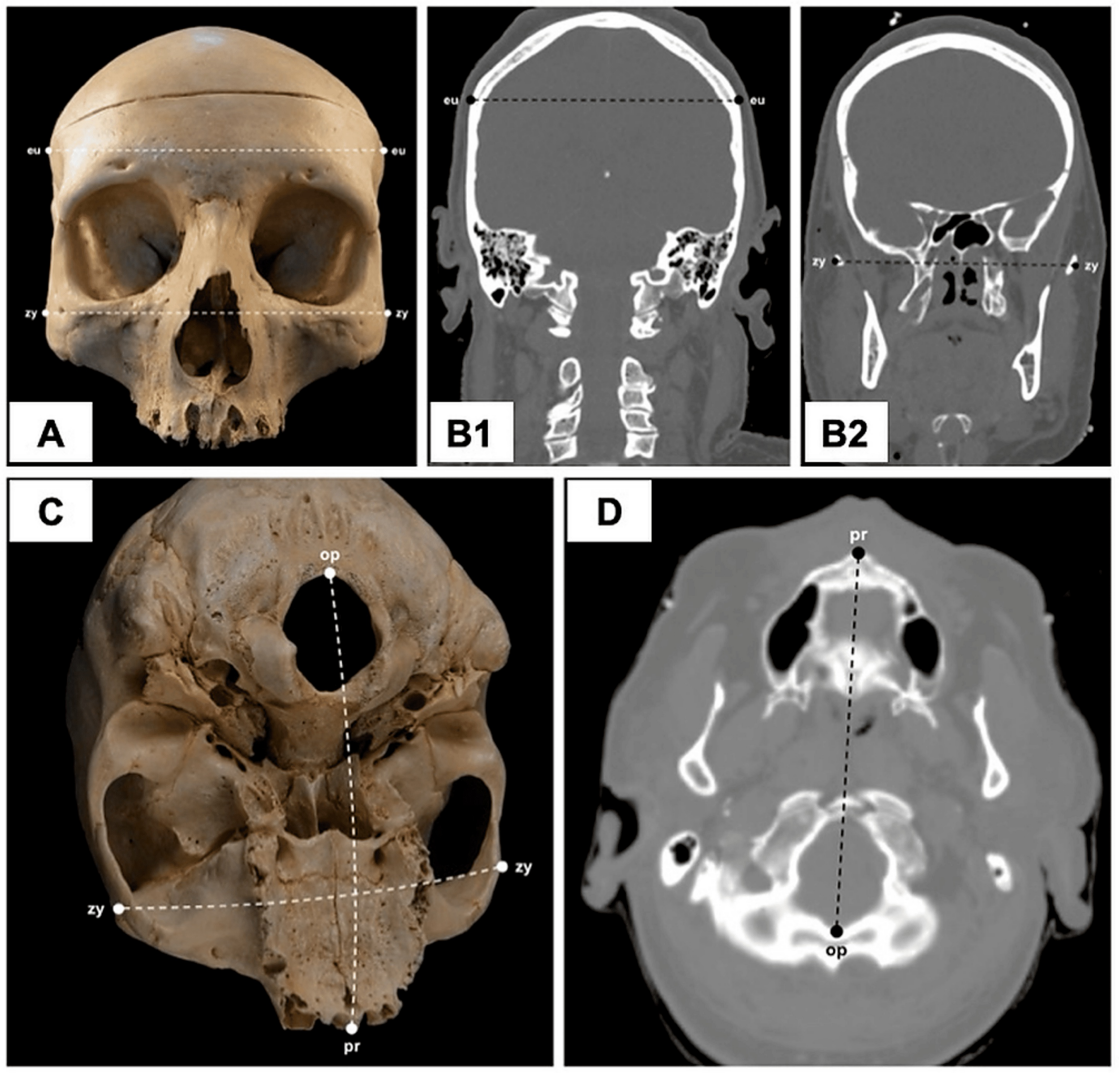
Craniometric variables assessed in dry skulls and CT examinations (A) Frontal dry-skull view showing maximum cranial breadth and bizygomatic breadth. (B1) Coronal CT image showing maximum cranial breadth. (B2) Coronal CT image showing bizygomatic breadth. (C) Inferior dry-skull view showing skull base length and bizygomatic breadth. (D) CT depiction of skull base length. CT: computed tomography; eu: euryon; op: opisthion; pr: prosthion; zy: zygion.

The recorded variables included four CEF, namely the parietal emissary foramen (PEF), sphenoidal emissary foramen (SEF), mastoid emissary foramen (MEF), and posterior condylar canal (PCC), together with selected constant cranial foramina, craniometric parameters, and foramen-specific topographic measurements. The constant cranial foramina comprised the foramen ovale, foramen spinosum, jugular foramen, carotid canal, hypoglossal canal, and foramen magnum. The craniometric parameters comprised skull base length, maximum cranial breadth, and bizygomatic breadth measured according to standard craniometric landmarks. Representative osteological and CT depictions of these craniometric measurements are provided in Figure 1.

Each foramen was first assessed for side-specific presence. Presence was recorded separately on the right and left sides and then categorized at the specimen level as absent, unilateral, or bilateral. For each present CEF, anteroposterior and transverse diameters were recorded as the primary linear morphometric measurements. These measurements were used to describe foraminal size and to calculate derived indices of shape, estimated surface area, and side-to-side variation.

Morphologic classification was based on the diameter ratio (DR), calculated as DR = major diameter/minor diameter. Values from 1.0 to 1.1 were classified as round, values greater than 1.1 and up to 2.0 as oval, and values greater than 2.0 as irregular. The estimated surface area was calculated assuming an elliptical outline, using the formula Area = π × (AP/2) × (TR/2), where AP and TR represent the anteroposterior and transverse diameters, respectively. Side-to-side variation in bilaterally present foramina was quantified using a dimensionless percentage-type asymmetry index (AI), calculated as AI = [(R − L)/((R + L)/2)] × 100, where R and L represent the right- and left-sided measurements, respectively. Values close to zero indicated relative symmetry, positive values indicated right-sided predominance, and negative values indicated left-sided predominance.

**Figure 2:**
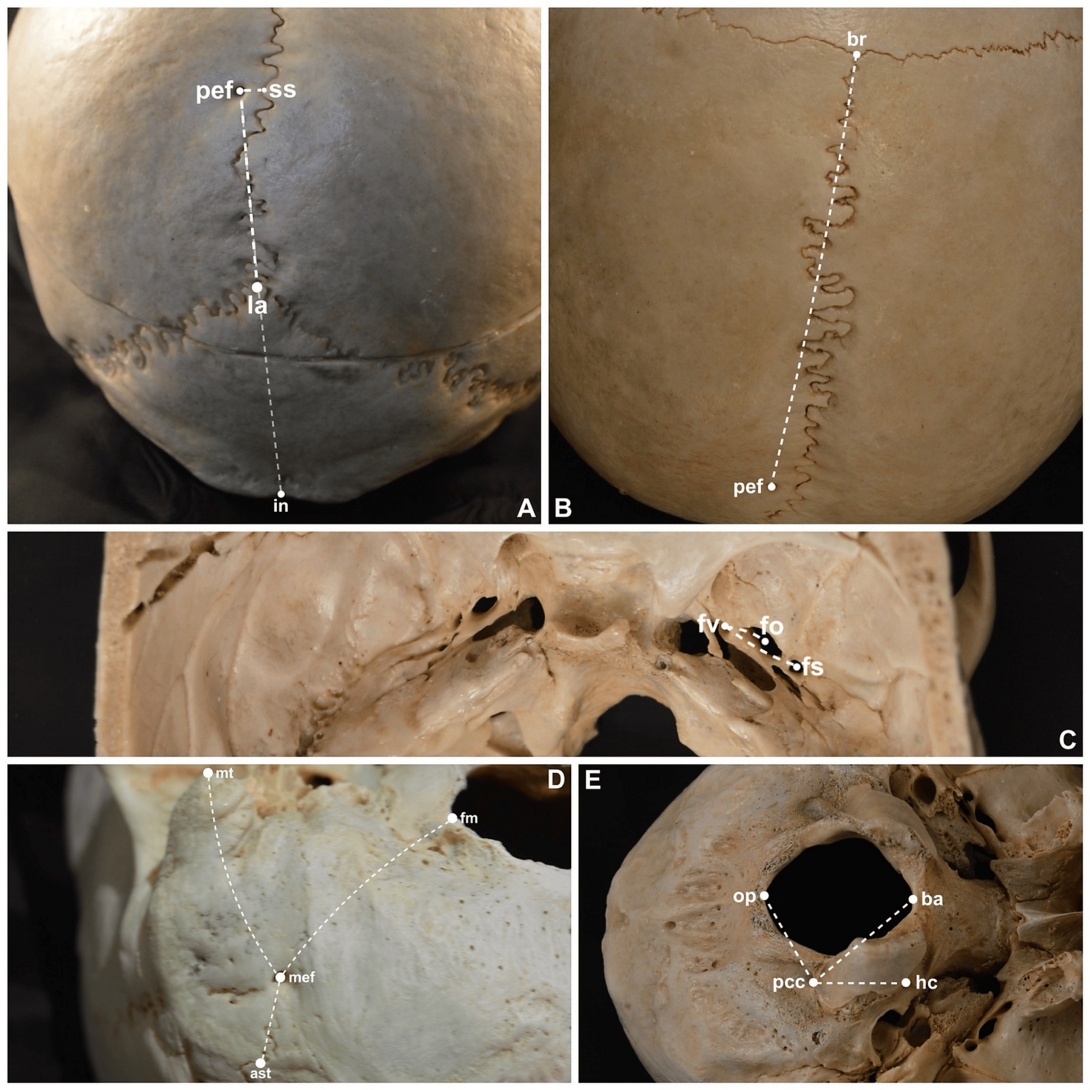
Foramen-specific topographic measurements Dashed lines indicate straight-line distances from each cranial emissary foramen to predefined anatomical landmarks or sutures. (A) Parietal emissary foramen distances from the sagittal suture, lambda, and inion. (B) Parietal emissary foramen distance from bregma. (C) Sphenoidal emissary foramen distances from the foramen ovale and foramen spinosum. (D) Mastoid emissary foramen distances from the mastoid tip, asterion, and foramen magnum. (E) Posterior condylar canal distances from the hypoglossal canal, basion, and opisthion. ast: asterion; ba: basion; br: bregma; fm: foramen magnum; fo: foramen ovale; fs: foramen spinosum; fv: foramen of Vesalius/sphenoidal emissary foramen; hc: hypoglossal canal; in: inion; la: lambda; mef: mastoid emissary foramen; mt: mastoid tip; op: opisthion; pcc: posterior condylar canal; pef: parietal emissary foramen; ss: sagittal suture.

Topographic measurements were used to describe the position of each CEF in relation to predefined regional anatomical landmarks. Measurements that could be consistently assessed on both dry skulls and CT examinations included the PEF-sagittal suture distance, SEF distances from the foramen ovale (FO) and foramen spinosum (FS), MEF distances from the mastoid tip and foramen magnum, and PCC distances from the hypoglossal canal, basion, and opisthion. Additional dry-skull measurements included PEF distances from bregma, lambda, inion, and the lambdoid suture, as well as MEF distance from the asterion. Representative illustrations of the foramen-specific topographic measurements are provided in Figure 2.

Further operational details on craniometric landmarks, derived morphometric indices, measurement rules, and foramen-specific topographic distances are available via the supplementary documentation referenced in the Appendix.

In accordance with the predefined measurement protocol, all eligible specimens were assessed using the same core anatomical variables, with modality-specific adaptations for direct osteological and CT-based evaluation. Dry skulls were assessed by direct visual inspection, with probing using a dental K-file or thin wire when foraminal patency was uncertain. Linear measurements were obtained with a high-precision digital caliper. CT examinations were evaluated on axial images, with multiplanar and three-dimensional reconstructions performed when required. Radiological measurements were obtained using Horos (Horos Project, Annapolis, USA) and 3D Slicer. 

Descriptive morphologic, morphometric, and topographic summaries were generated for the combined analytical sample. Potential modality-related differences between direct osteological and CT-based assessment were addressed analytically through the inclusion of specimen type in the exploratory modeling framework.

The primary anatomical outcome was the presence and distribution of each CEF. Secondary outcomes included laterality, morphology, morphometry, side-to-side variation, and topographic distances. Co-occurrence analyses and associations with selected cranial and skull-base variables, sex, and specimen type were considered exploratory.

Statistical analysis

All statistical analyses were performed using Stata 19.5 (StataCorp LLC, College Station, USA). Continuous variables were assessed for distribution using the Shapiro-Wilk test and visual inspection of histograms and Q-Q plots. Normally distributed variables were summarized as mean and standard deviation, whereas non-normally distributed variables were summarized as median and interquartile range. Categorical variables were reported as absolute and relative frequencies.

Interobserver reliability (IOR) was evaluated before the main analysis on a representative subset of 60 observations, comprising 30 dry skulls and 30 CT examinations. Two independent observers performed repeated measurements separately using the predefined measurement protocol. Agreement for continuous linear measurements was quantified using a two-way random-effects intraclass correlation coefficient (ICC) model for absolute agreement, based on single measurements. ICC values were interpreted according to established reporting thresholds [9].

Side-to-side comparisons of bilateral continuous measurements were performed using the paired Student’s t-test or the Wilcoxon signed-rank test, according to the distribution of right-left differences. Between-group comparisons by sex and specimen type were performed using the independent-samples Student’s t-test or Mann-Whitney U test for continuous variables and Pearson’s chi-square test or Fisher’s exact test for categorical variables, as appropriate.

Inferential analyses were exploratory. Logistic regression was used to assess the presence of foraminal expression and, among cases with foraminal expression, bilateral rather than unilateral expression. Pairwise co-occurrence between CEF was evaluated using contingency analyses, with adjusted logistic regression models fitted when appropriate. Linear regression was used to examine foramen-specific topographic distances as continuous outcomes. Candidate predictors were selected according to anatomical relevance and included sex, specimen type, selected craniometric parameters, and morphometric variables of anatomically related constant cranial foramina. Specimen type, defined as dry skull versus CT examination, was retained in the principal multivariable models to account for potential modality-related differences in anatomical assessment.

Continuous predictors were standardized before multivariable modeling to improve comparability of effect estimates. Results from logistic regression models were reported as odds ratios with 95% confidence intervals, and results from linear regression models were reported as regression coefficients with 95% confidence intervals. Because the inferential analyses were exploratory and hypothesis-generating, no formal adjustment for multiple comparisons was applied. Further details on predictor selection, collinearity assessment, model specification, robustness checks, supportive analyses, derived indices, and landmark definitions are available via the supplementary documentation referenced in the Appendix.

## Results

Sample characteristics, prevalence, and laterality

A total of 200 specimens were included in the final analysis, comprising 50 dry skulls (25.0%) and 150 CT examinations (75.0%). The sample comprised 105 male specimens (52.5%) and 95 female specimens (47.5%). Among the dry skull specimens, 27 were male and 23 were female; among the CT examinations, 78 were male and 72 were female. The PCC was present in 162 specimens (81.0%), the MEF in 157 specimens (78.5%), the PEF in 137 specimens (68.5%), and the SEF in 49 specimens (24.5%). Among specimens in which the corresponding foramen was present, bilateral expression was observed in 64 PEF cases (46.7%), 12 SEF cases (24.5%), 74 MEF cases (47.1%), and 73 PCC cases (45.1%). Among unilateral cases, right-sided occurrence was recorded in 35 PEF cases (47.9%), 25 SEF cases (67.6%), 38 MEF cases (45.8%), and 43 PCC cases (48.3%). Detailed prevalence, laterality, and unilateral side-wise distribution are presented in Table 1.

**Table 1:** Prevalence, laterality, and side-wise distribution of the examined cranial emissary foramina Values are presented as n (%). Percentages for foramen presence were calculated using the total sample as the denominator (n=200). Percentages for laterality (bilateral and unilateral presence) were calculated among specimens in which the corresponding foramen was present. Percentages for side-wise distribution (right- and left-sided occurrence) were calculated among unilateral cases only. CEF: cranial emissary foramina; PEF: parietal emissary foramen; SEF: sphenoidal emissary foramen; MEF: mastoid emissary foramen; PCC: posterior condylar canal.

CEF	Presence, n (%)	Laterality, n (%)		Side-wise distribution, n (%)	
		Bilateral	Unilateral	Right side	Left side
PEF	137 (68.5)	64 (46.7)	73 (53.3)	35 (47.9)	38 (52.1)
SEF	49 (24.5)	12 (24.5)	37 (75.5)	25 (67.6)	12 (32.4)
MEF	157 (78.5)	74 (47.1)	83 (52.9)	38 (45.8)	45 (54.2)
PCC	162 (81.0)	73 (45.1)	89 (54.9)	43 (48.3)	46 (51.7)

**Figure 3:**
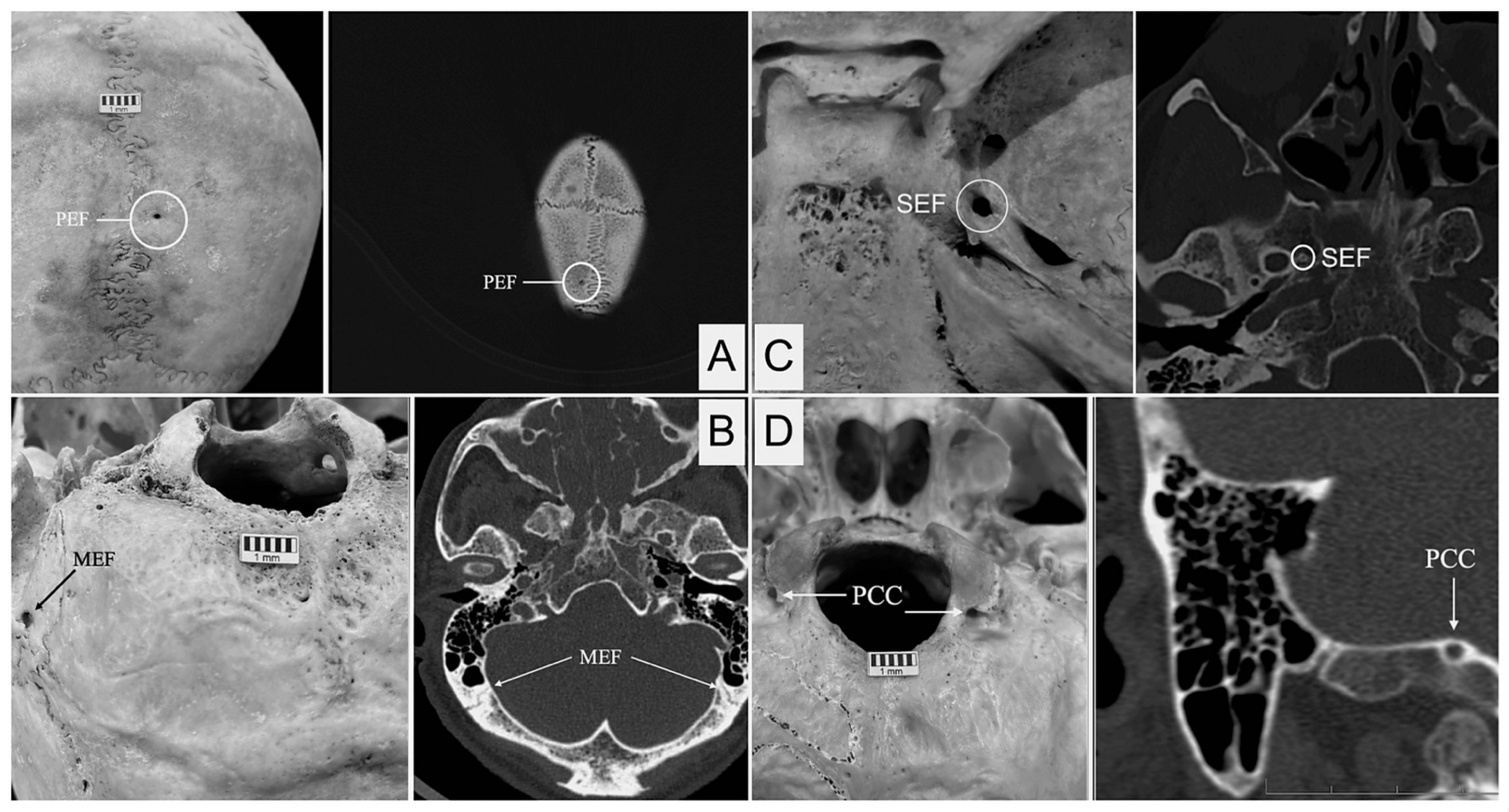
Representative dry-skull and CT appearances of the examined cranial emissary foramina Each foramen is shown in an osteological specimen and on the corresponding CT image. (A) Parietal emissary foramen. (B) Mastoid emissary foramen. (C) Sphenoidal emissary foramen. (D) Posterior condylar canal. CT: computed tomography; MEF: mastoid emissary foramen; PCC: posterior condylar canal; PEF: parietal emissary foramen; SEF: sphenoidal emissary foramen.

Examples of the examined CEF on dry-skull specimens and CT reconstructions are provided in Figure 3.

Interobserver reliability (IOR) for continuous measurements was high to excellent, with intraclass correlation coefficients (ICC) ranging from 0.850 to 0.910; all estimates were statistically significant (p<0.001).

Morphology, morphometry, and topographic anatomy

The pooled morphologic, morphometric, and topographic summaries from the combined dry-skull and CT-based sample are presented in Tables 2, 3. Morphologic classification showed a predominance of oval forms across the examined CEF. Overall morphometric values were greatest for the PCC and MEF and lowest for the SEF, while the estimated surface area was greatest for the PCC and lowest for the SEF.

For the PEF, the median side-averaged distances were 12.48 mm from the sagittal suture, 87.20 mm from bregma, 37.01 mm from lambda, 100.94 mm from inion, and 40.90 mm from the lambdoid suture. For the SEF, the median side-averaged distances were 2.40 mm from the foramen ovale (FO) and 11.12 mm from the foramen spinosum (FS). For the MEF, the median side-averaged distances were 27.94 mm from the mastoid tip, 37.58 mm from the foramen magnum, and 20.82 mm from the asterion. For the PCC, the median side-averaged distances were 8.10 mm from the hypoglossal canal, 27.77 mm from basion, and 29.63 mm from opisthion.

**Table 2:** Side-specific and side-averaged morphology and morphometry of the examined cranial emissary foramina The morphology column shows the most frequent morphological category and its frequency for each side. Side-averaged rows summarize numeric measurements only; morphology is not averaged across sides. The estimated area was calculated assuming an elliptical outline. Further methodological details on morphological classification, diameter-ratio typology, and derived morphometric calculations are provided in the supplementary documentation referenced in the Appendix. CEF: cranial emissary foramina; PEF: parietal emissary foramen; SEF: sphenoidal emissary foramen; MEF: mastoid emissary foramen; PCC: posterior condylar canal; DR: diameter ratio; AP: anteroposterior diameter; TR: transverse diameter; IQR: interquartile range.

CEF	Side	n	Morphology, n (%)	DR, median (IQR)	AP (mm), median (IQR)	TR (mm), median (IQR)	Area (mm^2^), median (IQR)
PEF	Left	102	Oval, 84 (82.4)	1.51 (1.23–1.76)	1.96 (1.61–2.26)	1.42 (1.24–1.71)	2.12 (1.69–2.75)
	Right	99	Oval, 73 (73.7)	1.50 (1.25–1.73)	1.97 (1.63–2.19)	1.32 (1.15–1.54)	1.90 (1.67–2.29)
	Side-averaged	137	-	1.51 (1.31–1.73)	1.97 (1.64–2.17)	1.41 (1.25–1.64)	2.08 (1.75–2.45)
SEF	Left	24	Oval, 19 (79.2)	1.25 (1.13–1.54)	1.48 (1.39–1.62)	1.26 (1.06–1.32)	1.46 (1.32–1.60)
	Right	37	Oval, 27 (73.0)	1.31 (1.11–1.45)	1.13 (0.99–1.26)	1.06 (0.91–1.17)	0.90 (0.80–1.02)
	Side-averaged	49	-	1.30 (1.13–1.45)	1.25 (1.10–1.45)	1.13 (0.94–1.29)	1.07 (0.91–1.35)
MEF	Left	119	Oval, 92 (77.3)	1.35 (1.16–1.64)	2.58 (2.21–2.98)	2.05 (1.67–2.45)	4.15 (3.11–5.26)
	Right	112	Oval, 79 (70.5)	1.34 (1.12–1.60)	2.26 (1.85–2.77)	2.32 (1.86–2.90)	4.11 (2.96–5.30)
	Side-averaged	157	-	1.35 (1.16–1.59)	2.48 (2.06–2.89)	2.16 (1.85–2.53)	4.07 (3.29–5.11)
PCC	Left	119	Oval, 88 (73.9)	1.30 (1.11–1.48)	3.29 (2.54–3.65)	2.99 (2.64–3.45)	7.46 (5.61–9.02)
	Right	116	Oval, 92 (79.3)	1.37 (1.18–1.70)	2.78 (2.12–3.51)	3.48 (2.77–3.66)	6.50 (5.51–8.64)
	Side-averaged	162	-	1.34 (1.15–1.52)	3.01 (2.51–3.53)	3.13 (2.78–3.61)	7.42 (5.81–8.73)

**Table 3:** Side-specific and side-averaged topographic distances of the examined cranial emissary foramina Topographic measurements represent straight-line distances from the external margin of the examined emissary foramen to the corresponding anatomical landmark or to the nearest portion of the relevant cranial suture. Side-specific measurements are presented separately for the left and right sides. Side-averaged values were calculated from the available side-specific measurements for each specimen; when both sides were available, the mean of the left- and right-sided measurements was used, whereas when only one side was available, the available side-specific measurement was retained. Measurements from bregma, lambda, inion, the lambdoid suture, and asterion were available only in dry skulls and were summarized descriptively. Further methodological details on landmark definitions and operational rules for topographic measurements are provided in the supplementary documentation referenced in the Appendix. CEF: cranial emissary foramina; PEF: parietal emissary foramen; SEF: sphenoidal emissary foramen; MEF: mastoid emissary foramen; PCC: posterior condylar canal; IQR: interquartile range.

CEF	Landmark	Side-averaged		Left side		Right side	
		n	Distance (mm), median (IQR)	n	Distance (mm), median (IQR)	n	Distance (mm), median (IQR)
PEF	Sagittal suture	128	12.48 (9.93–14.96)	91	12.89 (10.09–15.61)	90	12.15 (8.95–15.97)
	Bregma	30	87.20 (77.76–91.54)	23	89.22 (79.41–94.19)	18	78.31 (76.05–88.46)
	Lambda	30	37.01 (30.49–42.17)	23	36.66 (30.71–41.40)	18	34.56 (29.53–38.44)
	Inion	30	100.94 (91.04–110.51)	23	100.63 (89.65–113.21)	18	102.34 (94.54–108.04)
	Lambdoid suture	30	40.90 (35.64–49.36)	23	42.03 (35.77–48.58)	18	39.58 (35.64–49.13)
SEF	Foramen ovale	45	2.40 (2.07–2.79)	23	2.73 (2.23–3.03)	30	2.16 (1.97–2.57)
	Foramen spinosum	45	11.12 (10.57–11.97)	23	11.68 (10.87–12.37)	30	11.11 (10.29–11.62)
MEF	Mastoid tip	153	27.94 (25.58–31.17)	109	28.46 (24.85–31.61)	111	27.37 (24.23–29.91)
	Foramen magnum	153	37.58 (34.03–41.43)	109	38.01 (33.95–42.99)	111	37.84 (32.86–40.48)
	Asterion	39	20.82 (16.06–22.17)	29	21.53 (16.85–25.17)	25	18.50 (15.88–21.37)
PCC	Hypoglossal canal	154	8.10 (7.39–8.84)	110	8.29 (7.55–9.58)	107	7.69 (7.17–8.64)
	Basion	154	27.77 (25.96–29.42)	110	28.15 (25.76–30.33)	107	27.74 (26.02–29.60)
	Opisthion	154	29.63 (27.84–31.74)	110	30.02 (27.84–31.80)	107	29.59 (26.80–31.87)

Detailed tabulated summaries underlying the aggregated morphometric and topographic findings are provided in the supplementary documentation referenced in the Appendix. 

Exploratory association analyses

In the models for foraminal presence, no independent association with PEF was identified. SEF presence was associated with skull base length (OR=1.65, 95% CI: 1.05-2.60; p=0.032) and mean transverse diameter of the FS (OR=3.41, 95% CI: 1.11-10.46; p=0.032). MEF presence was inversely associated with the mean anteroposterior diameter of the hypoglossal canal (HC) (OR=0.61, 95% CI: 0.39-0.94; p=0.025), and PCC presence was associated with the mean transverse diameter of the HC (OR=2.24, 95% CI: 1.02-4.91; p=0.044).

For bilaterality, bilateral MEF presence was associated with female sex (OR=2.19, 95% CI: 1.07-4.48; p=0.031) and mean anteroposterior diameter of the jugular foramen (JF) (OR=1.67, 95% CI: 1.18-2.36; p=0.004). PCC bilaterality was inversely associated with cranial breadth (OR=0.63, 95% CI: 0.44-0.89; p=0.009). No independent association was identified for PEF bilaterality, and no multivariable model was retained for SEF bilaterality.

In the co-occurrence analysis, PCC presence was recorded in 44 specimens with an SEF (93.6%) and in 112 specimens without an SEF (73.2%). This association was statistically significant on chi-square testing [χ²(1)=8.73, p=0.003, Cramér’s V=0.21]. In the adjusted model, SEF presence was associated with PCC presence (OR=4.62, 95% CI: 1.33-16.07; p=0.016), and skull base length was retained (OR=1.63, 95% CI: 1.03-2.57; p=0.036).

For topographic distances, the PEF-sagittal suture distance was smaller in female specimens (β=-2.28, 95% CI: -3.75 to -0.80; p=0.003). PCC distance from the HC was associated with skull base length (β=0.46, 95% CI: 0.21-0.71; p<0.001), distance from basion with cranial breadth (β=0.70, 95% CI: 0.26-1.14; p=0.002), and distance from opisthion with sex (β=1.25, 95% CI: 0.26-2.23; p=0.013). No independent association was identified for the SEF or the MEF topographic distances. The corresponding significant estimates are summarized in Table 4.

**Table 4:** Significant exploratory associations involving cranial emissary foramen presence, bilaterality, co-occurrence, and topographic distances Values are derived from multivariable or adjusted exploratory models, and only statistically significant findings are shown. Logistic regression results are presented as odds ratios, and linear regression results are presented as β coefficients. For foramen-presence models, the outcome was presence versus absence of the corresponding emissary foramen. For bilaterality models, analyses were restricted to specimens in which the corresponding foramen was present, and the outcome was bilateral versus unilateral presence. The co-occurrence model evaluated SEF presence in relation to PCC presence, with skull base length retained in the adjusted model. Continuous predictors were standardized before regression modeling; therefore, effect estimates correspond to a one-standard-deviation increase in the predictor. Female sex was compared with male sex. Detailed model outputs underlying the summarized exploratory associations are provided in the supplementary documentation referenced in the Appendix. AP: anteroposterior diameter; CI: confidence interval; MEF: mastoid emissary foramen; OR: odds ratio; PCC: posterior condylar canal; PEF: parietal emissary foramen; SEF: sphenoidal emissary foramen; TR: transverse diameter.

Analysis type	Outcome	Associated predictor	Measure	Effect estimate	95% CI	p-value
Foramen presence	SEF presence	Skull base length	OR	1.65	1.05–2.60	0.032
Foramen presence	SEF presence	Foramen spinosum, mean TR	OR	3.41	1.11–10.46	0.032
Foramen presence	MEF presence	Hypoglossal canal, mean AP	OR	0.61	0.39–0.94	0.025
Foramen presence	PCC presence	Hypoglossal canal, mean TR	OR	2.24	1.02–4.91	0.044
Bilaterality	Bilateral MEF presence	Female sex	OR	2.19	1.07–4.48	0.031
Bilaterality	Bilateral MEF presence	Jugular foramen, mean AP	OR	1.67	1.18–2.36	0.004
Bilaterality	Bilateral PCC presence	Cranial breadth	OR	0.63	0.44–0.89	0.009
Co-occurrence	SEF presence	PCC presence	OR	4.62	1.33–16.07	0.016
Co-occurrence	SEF presence	Skull base length	OR	1.63	1.03–2.57	0.036
Topographic distance	PEF–sagittal suture distance	Female sex	β	-2.28	-3.75 to -0.80	0.003
Topographic distance	PCC–hypoglossal canal distance	Skull base length	β	0.46	0.21–0.71	<0.001
Topographic distance	PCC–basion distance	Cranial breadth	β	0.7	0.26–1.14	0.002
Topographic distance	PCC–opisthion distance	Female sex	β	1.25	0.26–2.23	0.013

Detailed model outputs underlying the summarized exploratory associations are provided in the supplementary documentation referenced in the Appendix.

## Discussion

The present study provides a combined anatomical and CT-based assessment of selected CEF in an adult Greek sample. The main finding is that the PEF, SEF, MEF, and PCC should not be interpreted as a homogeneous group of accessory cranial structures. Instead, each structure demonstrated a region-specific anatomical profile in terms of prevalence, laterality, morphology, morphometry, topographic relationships, co-occurrence patterns, and exploratory associations with adjacent cranial and skull-base variables.

Prevalence and co-occurrence

In the present sample, the PCC and MEF were the most frequently identified structures, whereas the SEF was the least frequent. The observed PEF frequency falls within the broad range reported in previous osteological and imaging studies [7,10], while the lower frequency of the SEF is consistent with studies describing this foramen as highly variable [11,12]. The common occurrence of the MEF and PCC is also in agreement with anatomical and radiological reports emphasizing the frequent presence of posterior skull-base emissary pathways [4,6].

These comparisons should be interpreted cautiously because prevalence estimates may vary according to population background, study material, imaging modality, and criteria for foraminal patency [11]. Thus, the main implication of the prevalence findings is that CEF should not be characterized by frequency alone, but should be interpreted in relation to their regional anatomy, mode of detection, and adjacent venous or osseous structures [5].

Co-occurrence analysis further supported a region-dependent interpretation. No generalized association pattern was observed across the examined foramina; the only consistent association involved the SEF and PCC. Because these structures are anatomically distant and belong to different skull-base regions, this finding should be regarded as exploratory rather than as evidence of direct anatomical or venous continuity.

Laterality and side-to-side variation

The laterality findings also support a region-dependent interpretation of CEF. The PEF, MEF, and PCC showed relatively balanced unilateral and bilateral expression, whereas the SEF was predominantly unilateral. This pattern is consistent with previous reports showing that laterality varies according to the foramen examined, population background, and method of assessment [13].

The PEF findings remained compatible with the broad variability reported in previous studies [7,14]. The predominantly unilateral pattern of the SEF should be interpreted cautiously because of the smaller number of positive cases and the known variability of this structure [12,15]. In contrast, the MEF and PCC were frequent, but their high prevalence was not accompanied by strong unilateral side predominance.

Side-to-side morphometric comparisons showed that bilateral presence did not necessarily imply bilateral symmetry. Significant differences were limited to selected measurements and did not follow a uniform directional pattern across all foramina. This supports the interpretation of presence, laterality, side-wise distribution, and side-to-side morphometry as related but distinct dimensions of anatomical variability [16].

Morphology, morphometry, and topographic anatomy

The morphologic and morphometric findings provide additional evidence that CEF vary beyond presence and laterality. These findings represent pooled descriptive estimates from the combined dry-skull and CT-based sample, consistent with the mixed anatomical-radiological design of the study. Specimen type was considered in the exploratory analytical framework to account for potential modality-related differences. Oval morphology predominated across the examined foramina; however, this common morphologic pattern did not correspond to a uniform size profile. The SEF showed the smallest dimensions, whereas the PCC showed the largest overall morphometric profile, reflecting the different cranial regions and osseous pathways represented by these structures.

Overall, the morphometric findings were consistent with previous anatomical and imaging studies, although direct numerical comparisons are limited by heterogeneous measurement protocols. The PEF dimensions were within the range reported in previous series [7,14], the SEF showed values consistent with the broad variability described in osteological and imaging studies [12,17], and the MEF and PCC measurements were broadly aligned with previous studies of the mastoid and posterior condylar regions [8,18-20]. These comparisons should be interpreted cautiously because many studies report a single maximum or mean diameter, whereas the present study recorded anteroposterior and transverse diameters and derived both the estimated area and diameter ratio.

Topographic anatomy provides an additional level of interpretation because the significance of a CEF depends on its relationship with adjacent landmarks as well as on its size. The PEF was related to the sagittal suture and posterior cranial vault [7,21], the SEF to the FO and FS [12,17], the MEF to the posterolateral skull base and mastoid region [18,20], and the PCC to the HC and occipital condylar region [8,19]. These topographic findings support a region-specific interpretation of CEF anatomy rather than a generalized anatomical pattern.

Associations with cranial morphology and adjacent foramina

The exploratory regression analyses did not support a single morphometric explanation for foraminal presence, bilateral expression, or topographic location. Instead, the observed associations were selective and differed according to the examined foramen and anatomical region, suggesting that CEF variation should be interpreted in relation to local cranial-vault or skull-base morphology rather than global cranial size alone [22].

No independent association was identified for PEF presence, whereas SEF presence was associated with skull base length and FS morphometry. This may reflect broader variation in middle cranial-base morphology, but it should not be interpreted as evidence of direct anatomical dependence [1]. Associations involving the MEF and PCC were observed for posterior skull-base morphometric variables. The PCC findings are regionally plausible given their relationship with the occipital condylar region, whereas the MEF findings should be interpreted more cautiously. Overall, these results are best regarded as exploratory anatomical correlations rather than causal or developmental associations.

Clinical implications and future directions

The clinical relevance of CEF depends on its regional anatomy and on whether an osseous foramen corresponds to a patent venous channel. EVs may contribute to collateral venous drainage and have been discussed as potential routes for infection, thrombosis, or tumor spread [23,24]. In surgical practice, prominent emissary channels may be relevant during skull-base, mastoid, and posterior fossa procedures because of potential bleeding or sinus-related complications [2,23]. In radiological practice, these foramina should be recognized as normal variants to avoid misinterpretation as fractures, lytic lesions, or pathological bone defects [25].

The practical implications are region-specific. The MEF and PCC are most relevant to posterior skull-base and posterior fossa assessment [26,27], the SEF to the FO, middle cranial fossa, and procedures involving transoval or trigeminal access routes [17], and the PEF to cranial-vault venous channels related to the superior sagittal sinus region [28]. Future studies should incorporate CT angiography, magnetic resonance (MR) venography, or surgical correlation to determine which osseous foramina correspond to functionally relevant emissary venous pathways [29]. Developmental and morphometric studies may also clarify whether the exploratory associations with cranial-base dimensions are reproducible anatomical patterns or sample-specific findings [30].

Strengths and limitations

The present study has several strengths. It used a mixed anatomical and CT-based design, combining direct osteological inspection with radiological assessment in a sample of 200 adult specimens. The examined foramina were evaluated under a common measurement framework, and the use of predefined landmarks, side-specific measurements, derived morphometric indices, and interobserver reliability assessment further supports the internal consistency of the measurement protocol.

Several limitations should nevertheless be acknowledged. First, the study sample was restricted to adult Greek specimens, and the findings should therefore be interpreted as population-specific anatomical data rather than as universally applicable prevalence or morphometric estimates. External validation in larger and more diverse samples is required before broader anatomical generalizations can be made.

Second, the mixed composition of the study material introduces unavoidable methodological heterogeneity. Dry skulls and CT examinations provide complementary but not identical information. Direct osteological inspection may facilitate identification of small external foraminal openings and surface landmarks, whereas CT permits radiological assessment of deeper canals and three-dimensional relationships. As a result, differences in visualization, detection thresholds, slice thickness, reconstruction parameters, and measurement conditions may have influenced prevalence estimates and morphometric values. Although specimen type was considered in the analytical framework, residual modality-related differences cannot be excluded. Accordingly, the pooled morphologic and morphometric summaries should be interpreted as combined anatomical-radiological descriptive estimates rather than as modality-specific reference values.

Third, not all topographic landmarks were available across both specimen types. Measurements involving bregma, lambda, inion, the lambdoid suture, and asterion were restricted to dry skulls and were therefore summarized descriptively. These dry-skull-only measurements were not included in the principal multivariable topographic regression models. Consequently, the topographic regression analyses do not cover the full set of measured landmark relationships and should be interpreted only with respect to landmarks that could be assessed consistently across the study material.

Fourth, the study evaluated osseous foramina and canals, not the corresponding venous structures directly. Therefore, the presence of a bony foramen or canal should not be assumed to indicate a patent, large-caliber, or hemodynamically relevant emissary vein. The study did not include CT angiography, MR venography, surgical findings, or clinical outcome data and therefore could not assess venous patency, flow direction, collateral drainage, bleeding risk, or clinical significance. The clinical implications discussed should therefore be understood as anatomical and radiological considerations rather than as directly demonstrated functional or clinical associations.

Fifth, the morphologic classification was based on an adapted diameter-ratio framework rather than on a classification system specifically validated for CEF. Although this approach improved reproducibility compared with purely visual description, it remains an operational method. Similarly, the estimated area was calculated assuming an elliptical outline and should be interpreted as an approximate composite descriptor of foraminal size rather than as a direct anatomical surface measurement.

Finally, the inferential analyses were exploratory. Some outcomes, particularly bilateral SEF, included relatively few cases, limiting model stability. Multiple anatomical variables and several regression models were evaluated, increasing the possibility of chance findings. Although collinearity diagnostics, model stability checks, and supportive analyses were used, residual confounding, model-selection effects, and sparse-data bias cannot be fully excluded. Accordingly, the observed associations, including the SEF-PCC co-occurrence and the foramen-specific regression findings, should be interpreted as hypothesis-generating anatomical associations requiring confirmation in independent anatomical and imaging cohorts.

## Conclusions

This mixed anatomical and CT-based study demonstrates that cranial emissary foramina are frequent but anatomically heterogeneous structures. The PCC and MEF were the most frequently identified foramina, followed by the PEF, whereas the SEF was less common and showed predominantly unilateral expression. Morphologic, morphometric, and topographic assessment demonstrated distinct regional profiles, with oval morphology predominating overall, smaller dimensions for the SEF, larger dimensions for the PCC, and foramen-specific relationships with adjacent cranial landmarks.

Exploratory analyses identified selective associations with craniometric variables, adjacent foraminal dimensions, and sex, depending on the foramen and outcome examined. The observed SEF-PCC co-occurrence should be interpreted as a hypothesis-generating finding rather than evidence of direct anatomical continuity or shared venous drainage. Overall, these findings support a region-specific approach to the assessment of CEF. Future studies using standardized definitions, consistent morphometric protocols, and vascular imaging are required to clarify the relationship between osseous foramina and functionally relevant emissary venous pathways.

## Appendices

The supplementary documentation accompanying this study consists of two files: Supplementary Methods and Supplementary Results. The Supplementary Methods file contains expanded protocol-level information on craniometric landmark definitions, topographic measurement procedures, derived morphometric indices, measurement rules, and exploratory statistical modeling. The Supplementary Results file contains detailed tabulated summaries corresponding to the aggregated descriptive, comparative, and regression-based findings presented in the main article.

This material is provided as extended methodological and analytical documentation accompanying the study.

The supplementary documentation is available at: 10.5281/zenodo.20314901
